# SankeyNetwork: A clear and concise visualization tool for bibliometric data

**DOI:** 10.1016/j.mex.2025.103379

**Published:** 2025-06-03

**Authors:** Sher-Wei Lim, Willy Chou, Lifan Chen

**Affiliations:** a Department of Neurosurgery, Chi-Mei Medical Center, Chiali, Tainan, Taiwan; b Department of Nursing, Min-Hwei College of Health Care Management, Tainan, Taiwan; c Department of Leisure and Sports Management, CTBC University, Tainan, Taiwan; d Department of Physical Medicine and Rehabilitation, Chiali Chi-Mei Hospital, Tainan 710, Taiwan; e Department of Nursing, Tainan Municipal An-Nan Hospital- China Medical University, Tainan, Taiwan

**Keywords:** Bibliometric analysis, Country collaboration, Co-word occurrence, Network, Gene Expression Analysis, Sankey diagram, Slope graphs, Performance sheet, Sankey-type diagrams as Network [SankeyNetwork]

## Abstract

This study proposes a novel framework to overcome the limitations of traditional bibliometric visualizations—such as co-word network charts—by integrating Sankey diagrams with author collaborations and co-word occurrences to better identify key contributors and themes. Analyzing 2252 articles published in the *Journal of METHODSX* (2020–2024), the study focuses on ten essential metadata elements commonly used in bibliometric evaluations, including country, institution, department, authorship, and keywords.

Three complementary approaches are introduced: (1) a summarized performance sheet to present key metrics across entities, (2) Sankey diagrams for streamlined cluster visualization using the Following-Leading Clustering Algorithm (FLCA), and (3) slope graphs to track temporal trends and research bursts.

Findings highlight the dominance of the United States, Symbiosis International in India, and author Fengxiang X Han, with the keyword “MODEL” emerging as most frequent. A 2020 article by Wondimagegn Mengist received the highest citation count (370). Slope graphs showed upward trends in four core elements over the past four years.

The study concludes that these methods provide clearer insights while reducing visual complexity, and recommends combining performance sheets, Sankey diagrams, and slope graphs in future bibliometric analyses to better detect hotspots and evolving research patterns.

• Sankey diagrams to enhance traditional bibliometric visualization methods.
• Analyzing 2252 articles from Journal of METHODSX (2020–2024) to highlight author collaborations.
• Key insights include the prominence of U.S., and Symbiosis International (India) in author collaborations.

## Graphical abstract

**Figure:**
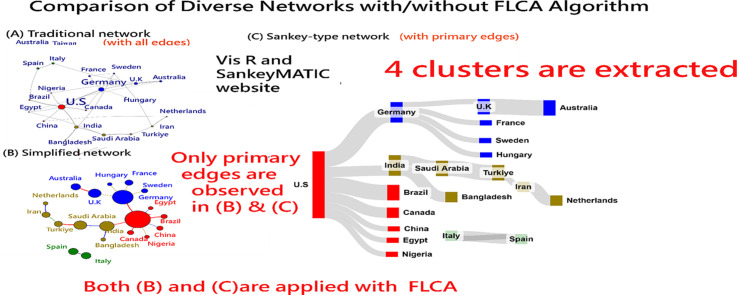

Specifications table

**Table:** 

Subject area	Mathematics and Statistics Economics, Econometrics and Finance Social Sciences Sciences Medical Sciences
More specific subject area	Visual representation
Name of your method	Sankey-type diagrams as Network [SankeyNetwork]
Name and reference of original method	[10] Daniel, D., & West-Mitchell, K. (2024). The Sankey diagram: An exploratory application of a data visualization tool. Transfusion, 64(6), 967–968. https://doi.org/10.1111/trf.17803
Resource availability	R code is available in: Author-made R-platform at https://www.raschonline.com/raschonline/cbp.asp?cbp=DDIfordrugdruginteractionB

## Background

Time-series data are crucial in economic analysis for identifying trends, cycles, and seasonality [1]. Despite the availability of various visualization tools, slope graphs outperform temporal bar-segment plots (TBSP) by enabling direct pairwise comparisons over time. Empirical evidence from previous work demonstrates improved clarity in detecting trend shifts and outliers when using slope graphs, especially in visualizing research bursts over successive years [[2], [3], [4]].

### Time-series analysis in bibliometrics

In bibliometric research, time-series data capture research bursts and evolving trends across elements such as keywords, affiliations, and citations [3,4]. These bursts signify surges in academic interest, marking the emergence of new fields or shifts in research priorities [2]. Although slope graphs provide a more intuitive method for visualizing these changes compared to conventional techniques, existing methods for extracting core elements for slope graphs remain underexplored [[2], [3], [4]].

### Addressing research gaps in traditional bibliometrics

Integrating advanced visualization and statistical approaches can enhance bibliometric analysis by addressing key challenges [5]: (1) **Identifying Core Elements**: Traditional TBSP methods rely on raw counts rather than statistical significance. The UMP model comprises three stages: (1) Univariate analysis (e.g., negative log10 p-value as the criterion of >1.3) to screen for high-variance elements, (2) Multivariate analysis (e.g., absolute log2 fold change as to >1.0) to identify underlying structures, and (3) Predictive analysis to assess trend trajectories. This model ensures more robust selection of key bibliometric indicators over time [3,4]; (2) **Key Performance Indicators (KPIs)**: Many bibliometric studies lack concise reporting, making it difficult to interpret findings efficiently. Focusing on ten essential metadata elements—including publication year, country, institution, department, authorship, and keywords—streamlines analysis (e.g., 42 Tables and Figures in this study) [6]; (3) **Detecting Trends and Research Bursts**: Understanding topic evolution over time requires clear visualization methods. Slope graphs reveal emerging hotspots and shifting research priorities more effectively than traditional visualizations, as addressed in Section 1.1; (4) **Simplified Cluster Analysis**: Traditional network charts often contain excessive edges(e.g., collaboration network [7]), making key author collaborations and co-word occurrences difficult to interpret.

### Innovative approaches to bibliometric analysis

To address these challenges, this study introduces three novel visualization strategies, particularly with a focus on cluster analysis using SankeyNetwork:

1. Summarized Performance Sheets – A concise reporting format focusing on ten key metadata elements to improve readability and interpretation.
2. Sankey Diagrams – A streamlined network visualization technique that prioritizes primary connections, reducing visual clutter [[8], [9], [10]].
3. Slope Graphs for Temporal Trends – A method for tracking research bursts and trend shifts over time, offering a more effective alternative to TBSP [3,4].

### Study objectives

This study verifies the hypothesis that integrating summarized performance sheets, Sankey diagrams, and slope graphs enhances bibliometric analysis, offering clearer insights while minimizing overly complex visualizations.

## Method details

### Data source

We retrieved metadata for 2252 articles published in the Journal of METHODSX from 2020 to 2024, as of January 22, 2025, using the Web of Science Core Collection (WoSCC) database. The metadata encompassed ten essential categories, as listed in Section 1.2.

All data are publicly available from WoSCC and are included in Appendix 1 without any participant identification information; thus, ethical approval was waived.

### Three steps with a focus on cluster analysis with SankeyNetwork to display

This study comprises four key components:

**Performance Sheet**: A summary of the top 10 elements within article entities, offering a concise alternative to the extensive graphs and tables commonly found in traditional bibliometric analyses.

**Cluster Analysis**: The Following-Leader Clustering Algorithm (FLCA) [11], which identifies the most influential link (leader-follower) for each node, thereby simplifying network complexity, was selected(i.e., only the most influential link per node are retained, thus reducing network density and enhancing visual clarity). This is particularly effective in Sankey diagrams, where interpretability of directional flows is crucial.. Sankey diagrams were drawn with scripts [[12], [13], [14]] in R [15].

**Gene Expression Analysis (GEA)**: Application of GEA [16] to extract significant article elements in identifying core elements through univariate and multivariate analyses using volcano plot and heatmap [17].

**Visualizations**: Creation of various visualizations, including performance sheet, Sankey diagrams, and slope graphs based on observed element counts in specific years.

#### Performance sheet

Traditional bibliometric analyses often employ numerous graphs and tables to identify prolific and influential entities [3,4,7]. A concise performance sheet summarizing the top 10 elements across ten article entities is urgently required.. This study introduces a unique performance sheet that includes the distribution of publication counts for first and corresponding authors (FP and RP, respectively), along with the h-index [18], providing a clearer overview of key contributors and their impact.

The dominance strength was evaluated using the Absolute Advantage Coefficient (AAC), calculated via Eqs. (1) and (2). For example, if the top three publication counts are 60, 30, and 15, then γ = (60/30)/(30/15) = 1, and AAC = 1 / (1 + 1) = 0.5. An AAC > 0.7 denotes strong dominance of the top-ranked element.

(1) AAC=γ/(1+γ),

(2) γ=(r1/r2)/(r2/r3),

where r1, r2, and r3 are the top three counts in modules. An AAC greater than 0.7 indicates a significant dominance effect of the top element against the next two.

Research hotspots and emerging trends are central themes in bibliometrics, yet no studies have employed GEA [16] on two distinct stages(e.g., 2020 to 2022 and 2023 to 2024 in this study) to identify research frontiers in the literature. Therefore, applying differential expression analysis (DEA) is crucial for effectively exploring these research frontiers.

#### Sankey diagrams

**Fig. 1:**
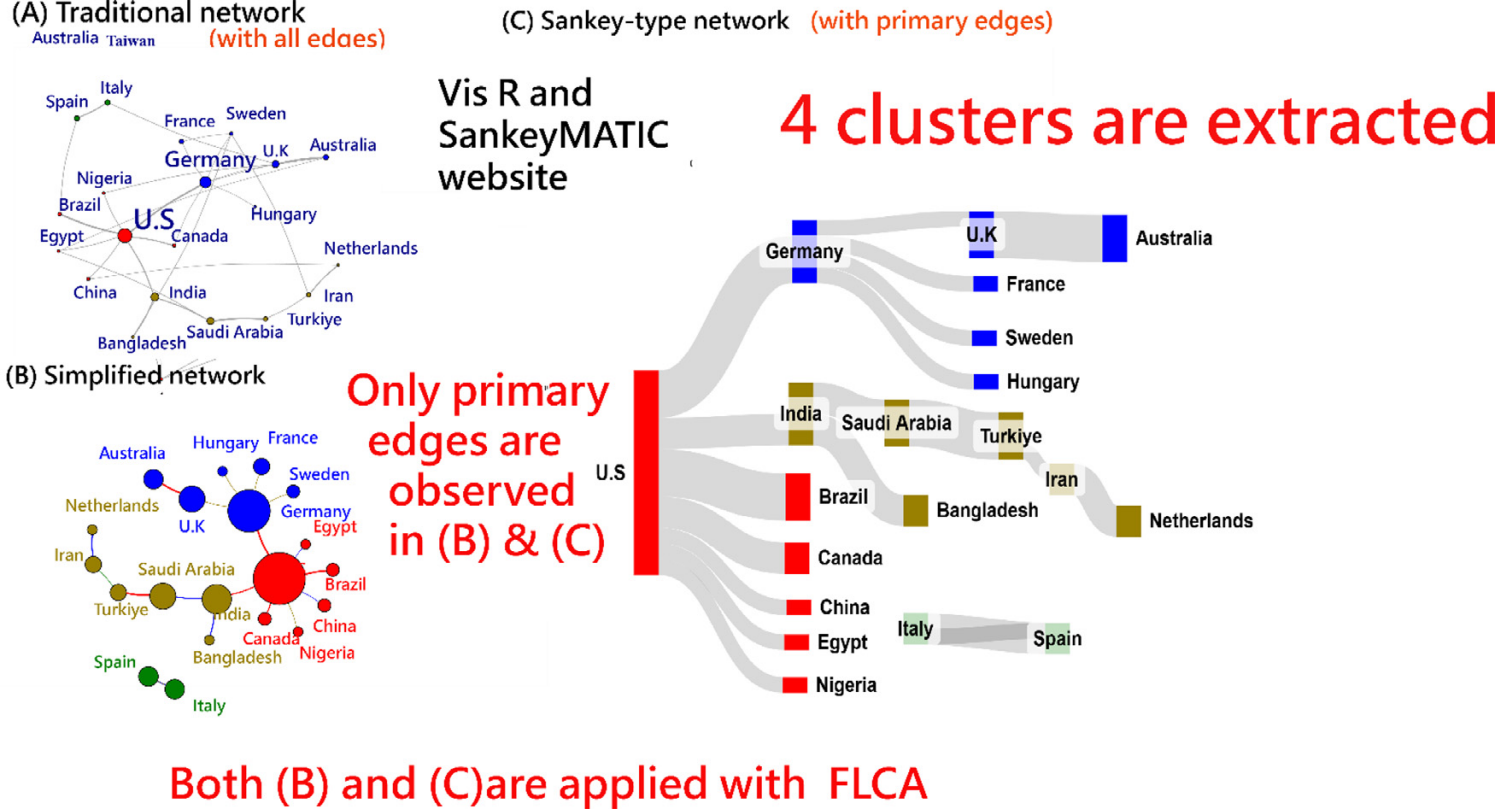
Networks in comparison with three types of visualizations.

Fig. 1 based on top 20 counties with most number of publications compares three types of network visualizations used in cluster analysis to simplify and enhance the clarity of relationships in country-based author collaborations for the Journal of METHODSX since 2020:

(A) **Network with Many Edges**: Displays all connections between countries, leading to visual clutter and making it difficult to interpret collaborations clearly.
(B) **Network with Primary Edges**: Highlights only the most significant edge (primary connection) for each node, reducing complexity and improving readability using FLCA [11].
(C) **Sankey Diagram with Primary Edges**: Uses a flow-based representation to emphasize primary relationships while maintaining a clear and structured view of collaborations between countries through FLCA [11] and Sankeymatic software [[12], [13], [14]]. Node size corresponds to publication count; edge thickness represents the strength of collaboration or co-occurrence. Colors indicate distinct clusters generated by FLCA. The left-to-right layout reflects the rank ordering of node prominence.

This approach in panel C of Fig. 1 simplifies network analysis and effectively communicates key relationships in a visually intuitive manner and highlights the major collaborations between countries (e.g., The US has more number of articles and collaborations with other counties in publications).

#### DEA in GEA to research articles

Differential Expression Analysis (DEA) is a crucial step in GEA that identifies genes with significantly different expression levels between distinct conditions, such as healthy vs. diseased states, treated vs. untreated samples, or different developmental stages. In this study, DEA is used analogously to compare element counts in publications across two phases, aiming to identify article elements (*n* = 65, derived from the top 10 elements in each entity on the performance sheet) that exhibit statistically significant changes in count.

Common tools for DEA include DESeq2, edgeR, and limma in R [15,17], which offer various statistical models and tests, such as negative binomial distributions (DESeq2, edgeR) or linear models for microarray data (limma). Given the large number of article elements tested simultaneously, multiple comparison corrections, such as the Benjamini-Hochberg procedure, are applied to control the false discovery rate (FDR) [21].

Five visualizations were employed to display significant changes, including performance sheet, volcano plots, heatmaps for significant elements, Sankey-type networks with thematic (or collaborative) maps [22,23], and slope graphs [24,25].

### R platform for visuals using SankeyNetwork

It is encouraged to use visual codes provided beneath the Figure legends on the R platform [15], with a focus on the Sankey diagrams in R script [15], by utilizing a copy-paste approach to create the visuals described in Sections 2.2.1–2.2.3.

The SankeyNetwork was implemented in this study using SankeyMATIC [12], incorporating links [14,26,27] to represent edges, counts, and temporal data, delt with FLCA algorithm [11]. An MP4 video [13] demonstrates these scenarios, with input CSV files containing two or more columns, such as first and corresponding authors, multi-authors, and keywords.

### Statistical analysis

Statistical analysis was conducted using R [15], incorporating |log10P| and negative logFC calculations with the limma packag. Visualizations in R created on the R platform [15] were introduced to verify the hypothesis that employing three distinct approaches—summarized performance sheets, Sankey-type networks, and slope graphs—will provide unique and innovative insights into bibliometric data when compared to the tradition [3,4]. Detailed instructions on generating visuals in R are provided in Appendix 2.

## Method validation

### Performance sheet

**Fig. 2:**
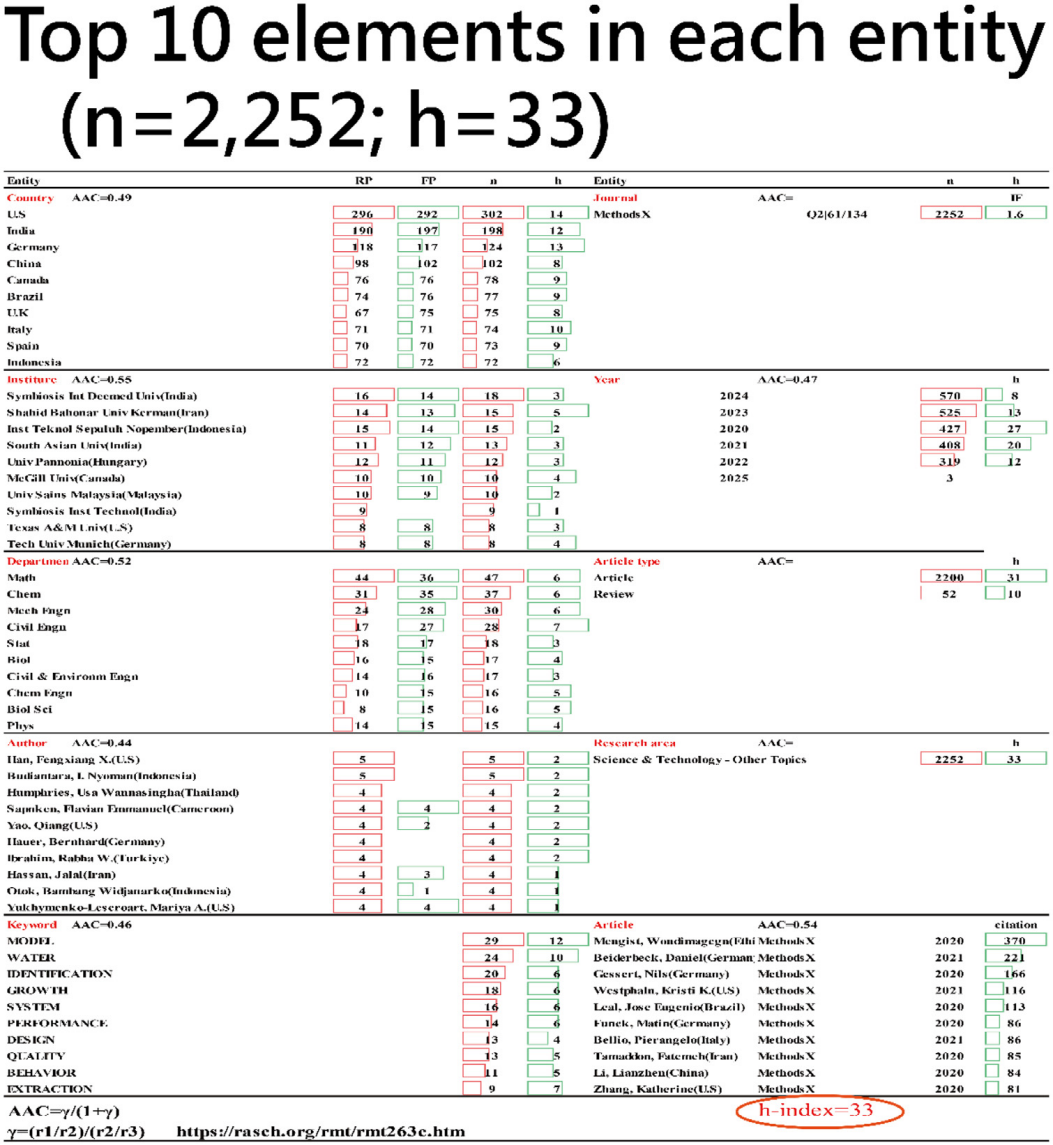
Summarized performance sheet for article elements in METHODSX since 2020.

Fig. 2 presents a performance sheet summarizing key contributors and counts across ten article entities in METHODSX, utilizing metrics such as publications by corresponding authors (RP), first authors (FP), h-index [18], and AAC [19,20] values. Overall Statistics include total articles analyzed: 2252 and overall h-index: 33. A summary of the contents extracted from the performance sheet with key findings in Fig. 2 is presented below: Key findings include the prominence of United States, Symbiosis International (Deemed University) in India, the Department of Mathematics, author Fengxiang X Han from United States, and the keyword "MODEL" in publications. The highest citation count (370) was for a 2020 article by Wondimagegn Mengist from Ethiopia. All AACs for the top elements across the 10 entities are below 0.70, indicating the absence of any significantly dominant roles in this study.

### Sankey-type networks for author collaborations and co-keyword occurrences

**Fig. 3:**
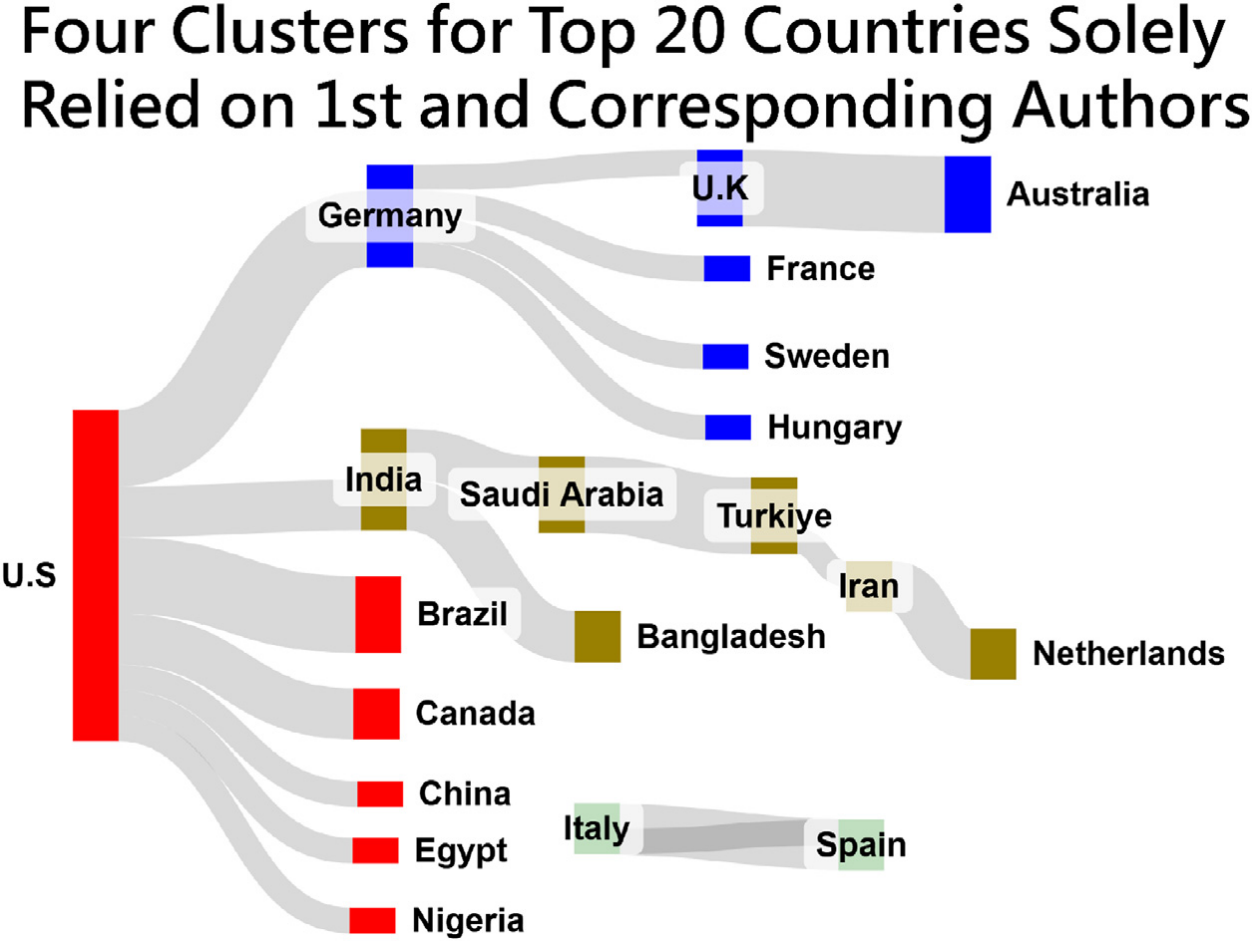
Top 20 country-based author collaborations(colored by clusters and sized by edges).

Fig. 3 through 5 showcase four Sankey diagrams summarizing author collaborations and co-keyword occurrences in METHODSX since 2020, all of which represent leaders by edges with FLCA algorithm [11, instead of that by counts, as shown in Appendix 3. It is clear to identify the author collaborations by country, institute, and occurrences by co-keyword, leading by the US, Symbiosis International (Deemed University) in India, and Model, respectively.

Moreover, it is worth noting that the Sankey diagram visually represents the connections between these keywords in Fig. 5, showing how they cluster under the three major research themes, including (1) model building(Highlighted in red), (2) water treatment (Highlighted in blue), and (3) management performance (Highlighted in brown). The thickness of the links indicates the strength of associations between different terms, and color coding differentiates between the thematic categories.

Accordingly, these diagrams in Fig. 3 through 5 simplify complex relationships, making it easier to identify key patterns and partnerships in research and publication networks: blocks are sized by the number of edges colored by clusters using FLCA [11], and located by counts observed in networks from the left to the right sides.

### DEA in GEA applied to research articles

The selected thresholds capture significant surges in element frequency, reflecting emerging or declining bibliometric trends between two defined phases (2020–2022 vs. 2023–2024). Significant elements (*n* = 4) were identified between the two phases using two criteria(i.e., |log10(p-value)| > 1.3 and absolute log2 fold change > 1.0), following standards in gene expression studies. These thresholds ensure statistical significance in identifying bibliometric element shifts across time phases, as depicted in the volcano plot (top of Fig. 6).

Frequency counts for the top 4 elements reveal clear contrasts between the two groups (Phases I and II shown in columns) and features of up-regulation (bottom of Fig. 6). Notably, all of those 4 elements have significantly increased.

### Slope graphs highlighting spots and trends

**Fig. 4:**
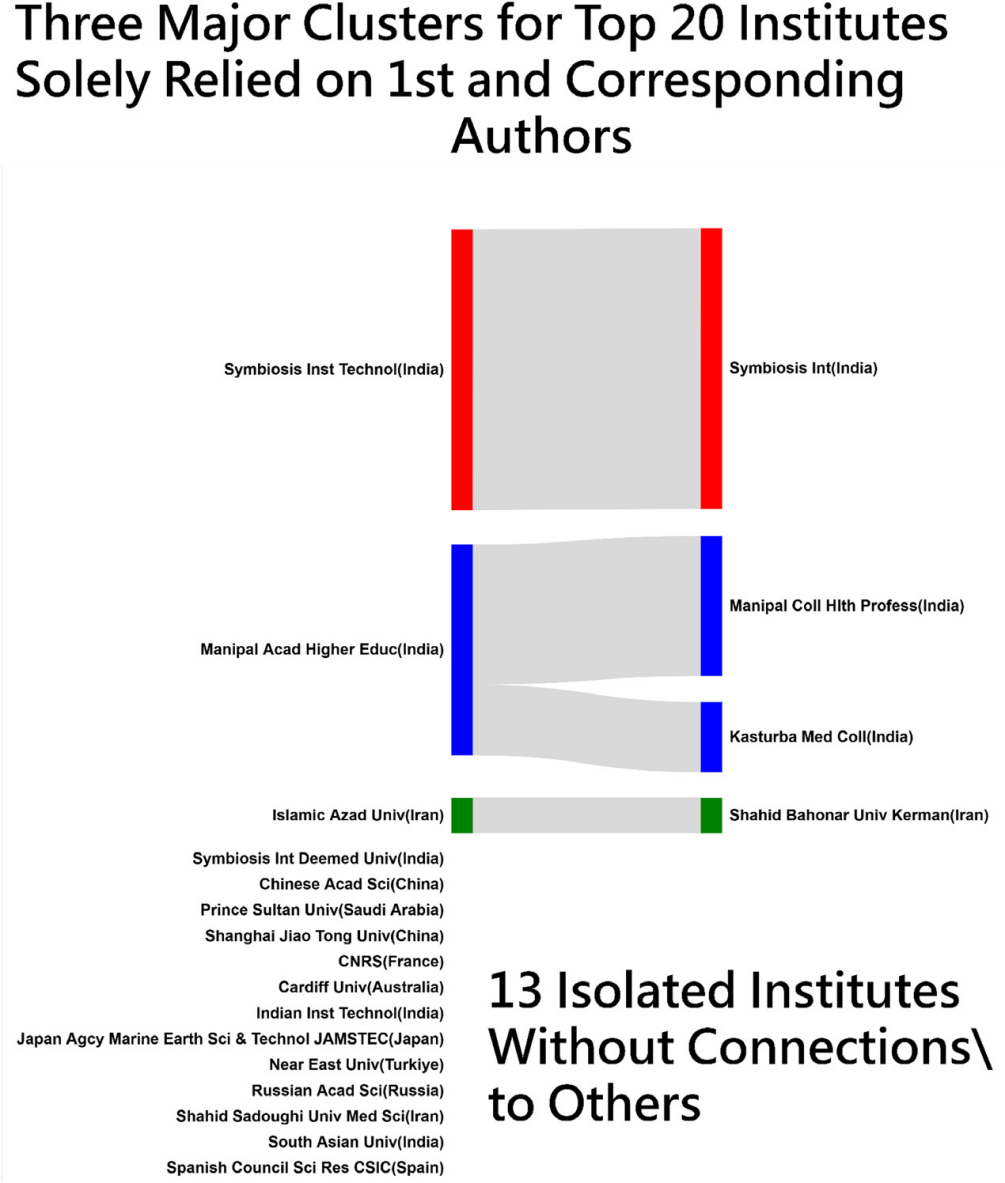
Top 20 institute-based author collaborations(colored by clusters and sized by edges).

Fig. 7 displays slope graphs for 4 core article elements, revealing one cluster only based on data correlations. Over the past four years, all elements have shown an upward trend. Compared to traditional burst-spot plot generated by CiteSpace software [28], slope graphs offer additional insights, including burst strength, duration of bursts for each element, and growth patterns (Fig. 4).

**Fig. 5:**
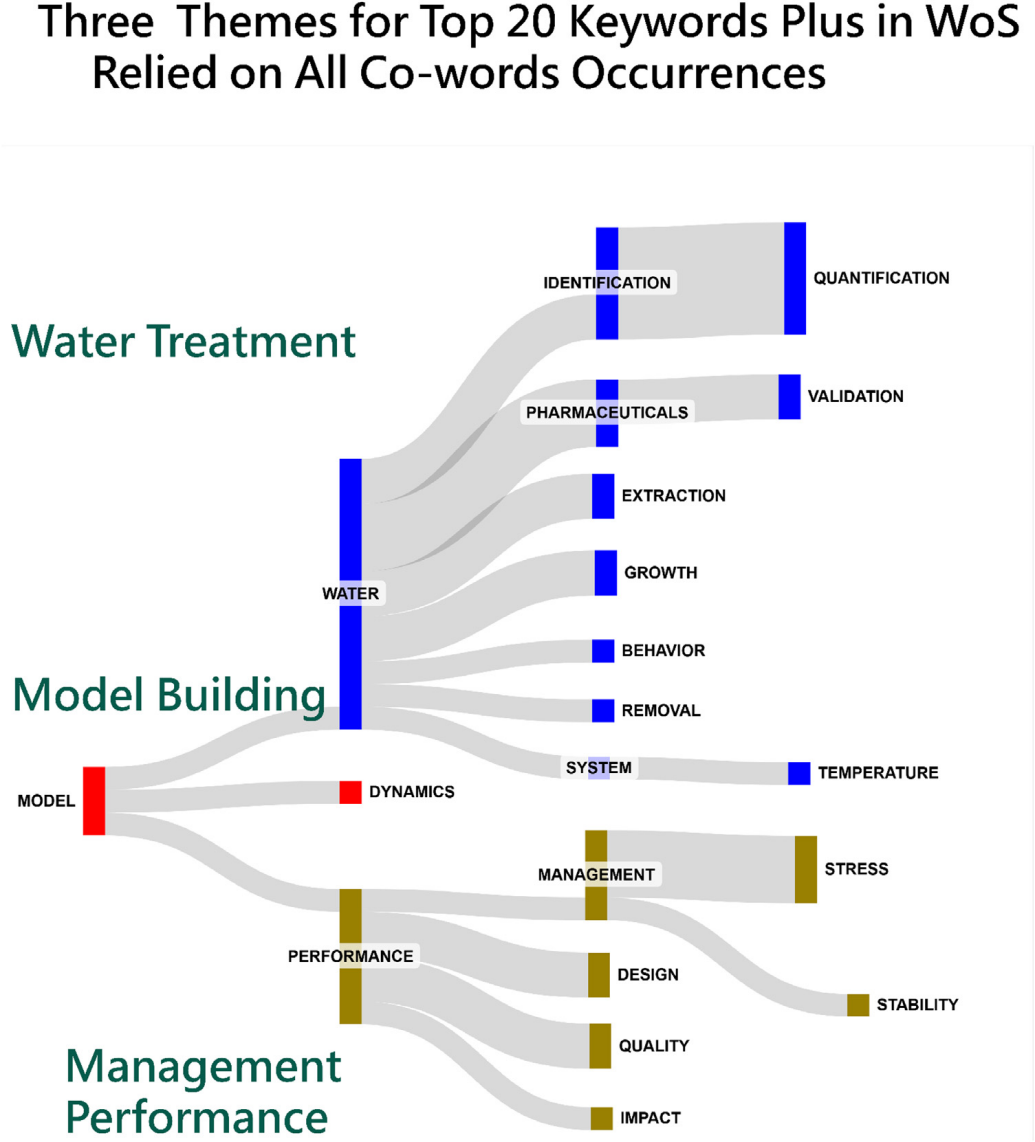
Coword analysis for top 20 keywords plus in Web of Science [29] (colored by clusters and sized by edges).

**Fig. 6:**
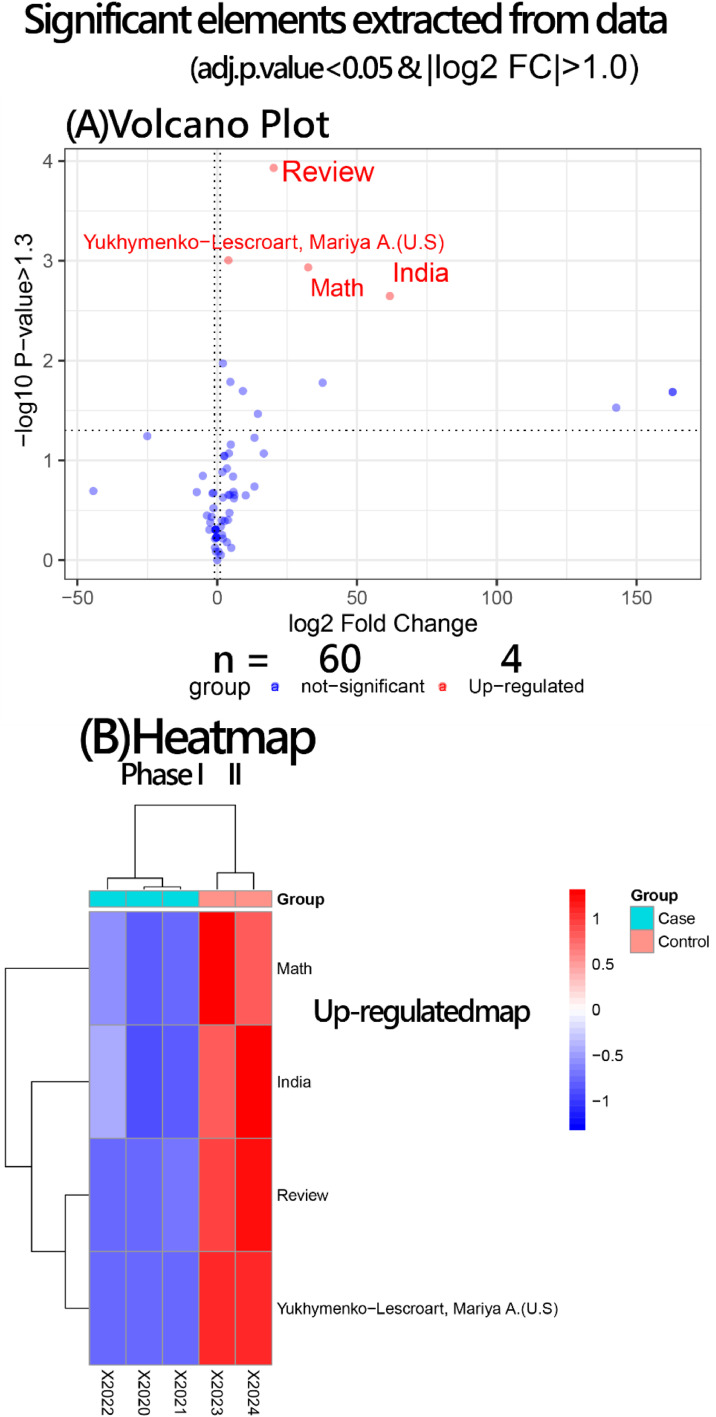
Significant elements with growth by count over years.

**Fig. 7:**
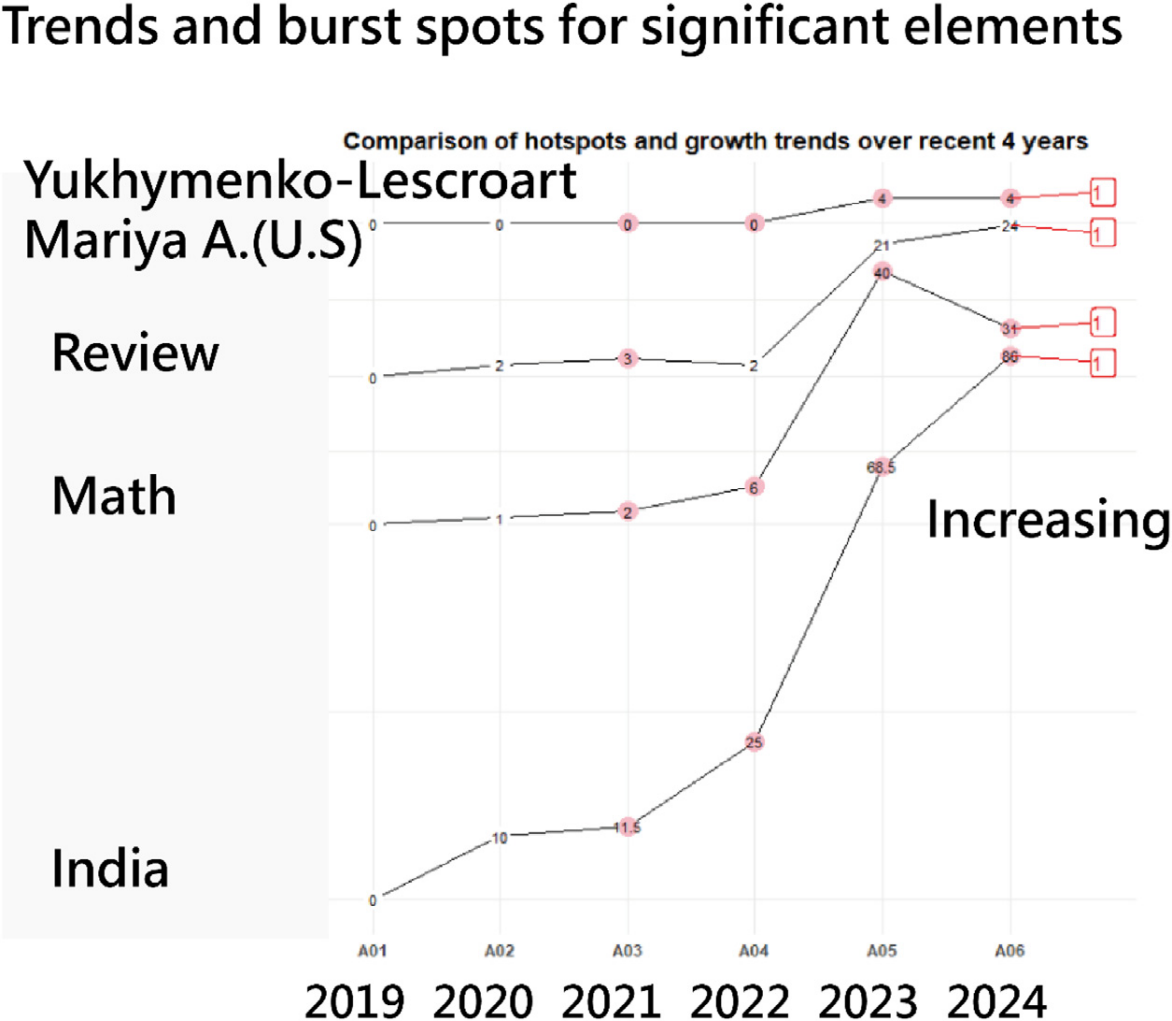
Slope graphs for significant elements(revealing one cluster only based on data correlations in one pink color; Over the past four years, all elements have shown an upward trend denoted by 1 at the right-hand side).

**Fig. 8:**
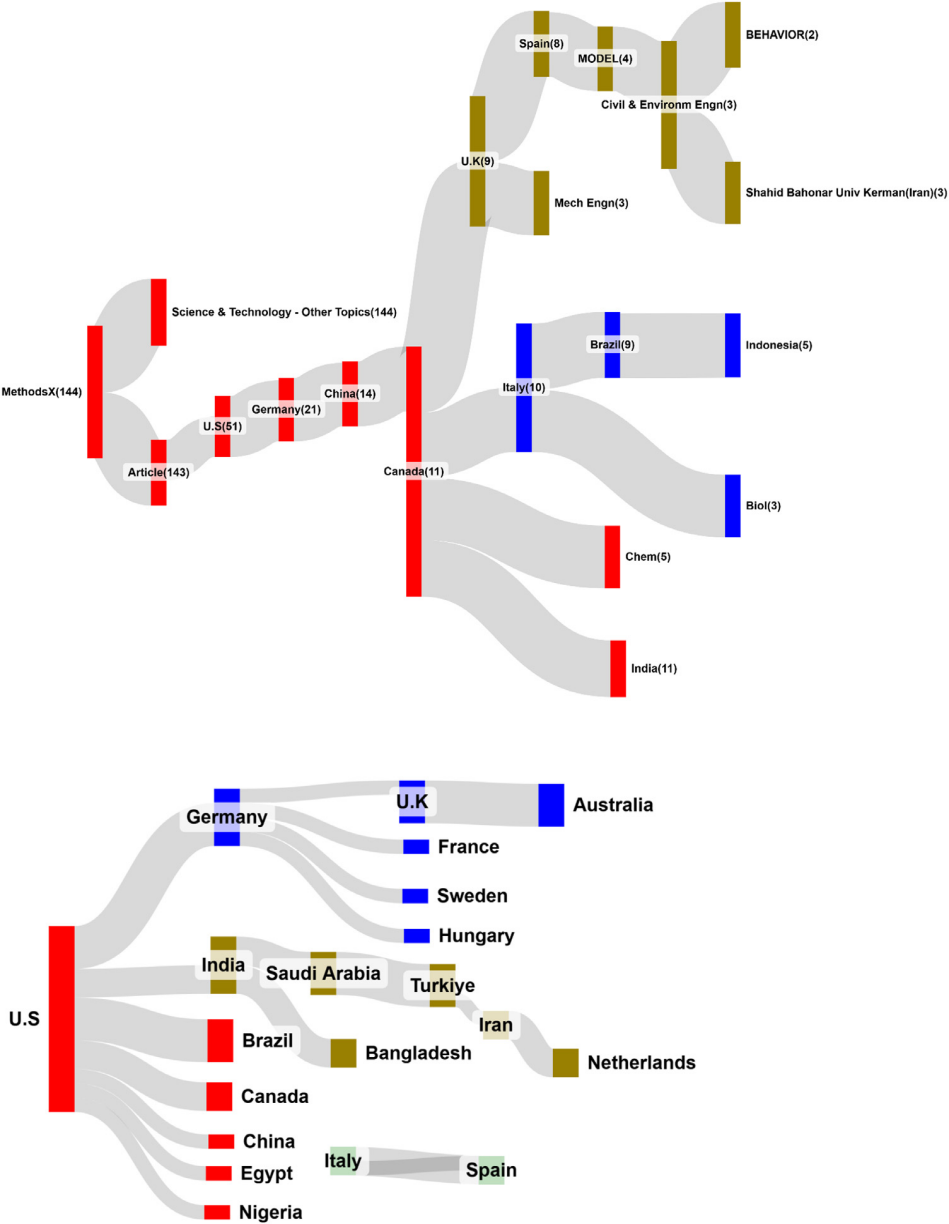
Sankey diagrams for candidate elements by data [27,32,33] (colored by clusters and sized by edges).

Sankey diagram for 65 candidate element is shown in Fig. 8, with temporal data [27,32,33]. It can be seen that a Sankey diagram visualizes keyword associations in MethodsX research. It links countries, institutions, disciplines, and themes such as performance, model, behavior, and countries, highlighting collaborations(or co-occurrences) and research focuses across various academic fields and geographic regions, with clusters by color.

### Illustration of visuals on R platform

Visuals can be generated using R scripts provided in this study. By copying the code into the input box at the top of the R-platform interface (or by using the provided links [14,26,27,32,33] with csv files), readers can select the desired visual type, and the corresponding R code will be generated in the output box. This R script can then be executed within the R environment. A basic understanding of R is required, including familiarity with predefined element labels in the script and adjusting parameters. Table 1 summarizes the strengths of Sankey, slope graphs, and performance sheets in bibliometrics.

**Table 1:** Summary comparing the strengths with Sankey, slope graphs, and performance sheets in bibliometrics.

Item	Visualization Method	Purpose	Key Output	Distinct Feature
1	Performance Sheet	Overview of top entities	Top 10 lists by category	Includes AAC and h-index
2	Sankey Diagrams	Cluster relationships	Directed edges and node flows	Simplified using FLCA
3	Slope Graphs	Temporal trend analysis	Research bursts and growth patterns	Easier interpretation than TBSP

## Limitations

Despite the advantages of slope graphs, Sankey diagrams, and summarized performance sheets, several limitations remain:

• **Limited Replicability of Slope Graphs** – Although slope graphs effectively identify research bursts and trends, their underutilization in bibliometric studies persists. Future research should ensure that R scripts [27] are provided for reproducibility and broader adoption.
• **Challenges in Core Element Extraction** – Existing methods lack a structured approach for extracting key bibliometric elements. Researchers must apply univariate, multivariate, and predictive analyses to refine element selection, ensuring meaningful trend identification.
• **Complexity in Network Visualizations** – Traditional bibliometric network charts often display excessive edges, making author collaborations and co-word occurrences difficult to interpret due to visual clutter. The provided R scripts [14,26,27,32,33] require more user-friendly interfaces for broader usability and validation.
• **Limitations of Existing Bibliometric Tools** – Platforms like Bibliometrix R [30], VOSviewer [31], and CiteSpace [31] struggle with multi-edge networks and country-level analyses, necessitating Sankey diagrams for improved clarity. However, their effectiveness in large-scale bibliometric analyses requires further verification by independent researchers.
• **Scalability of Sankey Diagrams** – While Sankey diagrams enhance network structure and readability, their application to large datasets remains untested. Additional validation is needed to assess their utility in highly complex bibliometric networks.
• SankeyNetwork was demonstrated using two types of illustrations: edges-based and temporal data-based visualizations [14,27]. While a count-based Sankey diagram is provided in Appendix 3, the impact of leading elements on cluster formation using FLCA [32] remains unexplored. Due to space limitations, a detailed analysis of this aspect was not included in this manuscript and should be addressed in future research.

Future research should focus on improving accessibility of visualization tools, enhancing automation for large-scale bibliometric studies, and ensuring reproducibility through shared R scripts.

## Ethics statements

Our study did not involve human subjects, animal experiments, or data collected from social media platforms. The work presented is based on the development and application of computational algorithms for bibliometric visualization using publicly available datasets from Web of Science. All data used were obtained from openly accessible sources, and no personally identifiable or sensitive information was involved. The methodologies applied comply with ethical research standards and adhere to the principles of transparency, reproducibility, and responsible data handling.

## Data availability

The data are public and available in Appendices:

Appendix 1: XLS(study data)_

Appendix 2: PDF (instruction on how to conduct this study)

Appendix 3: PNG (country-based author collaborations by count [26])

## CRediT authorship contribution statement

**Sher-Wei Lim:** Conceptualization, Methodology, Formal analysis, Writing – original draft. **Willy Chou:** Methodology, Data curation, Investigation, Writing – review & editing. **Lifan Chen:** Conceptualization, Supervision, Validation, Data curation, Writing – review & editing.

## Declaration of competing interest

The authors declare that they have no known competing financial interests or personal relationships that could have appeared to influence the work reported in this paper.

## Appendix Supplementary materials

Image, image 1

Image, application 2

Image, application 3

Image, video 4
