# Supplementary figures and images for: SankeyNetwork: A clear and concise visualization tool for bibliometric data
Source: MethodsX. 2025 Jun 3;14:103379. doi: 10.1016/j.mex.2025.103379 (PMC12179738; doi:10.1016/j.mex.2025.103379)

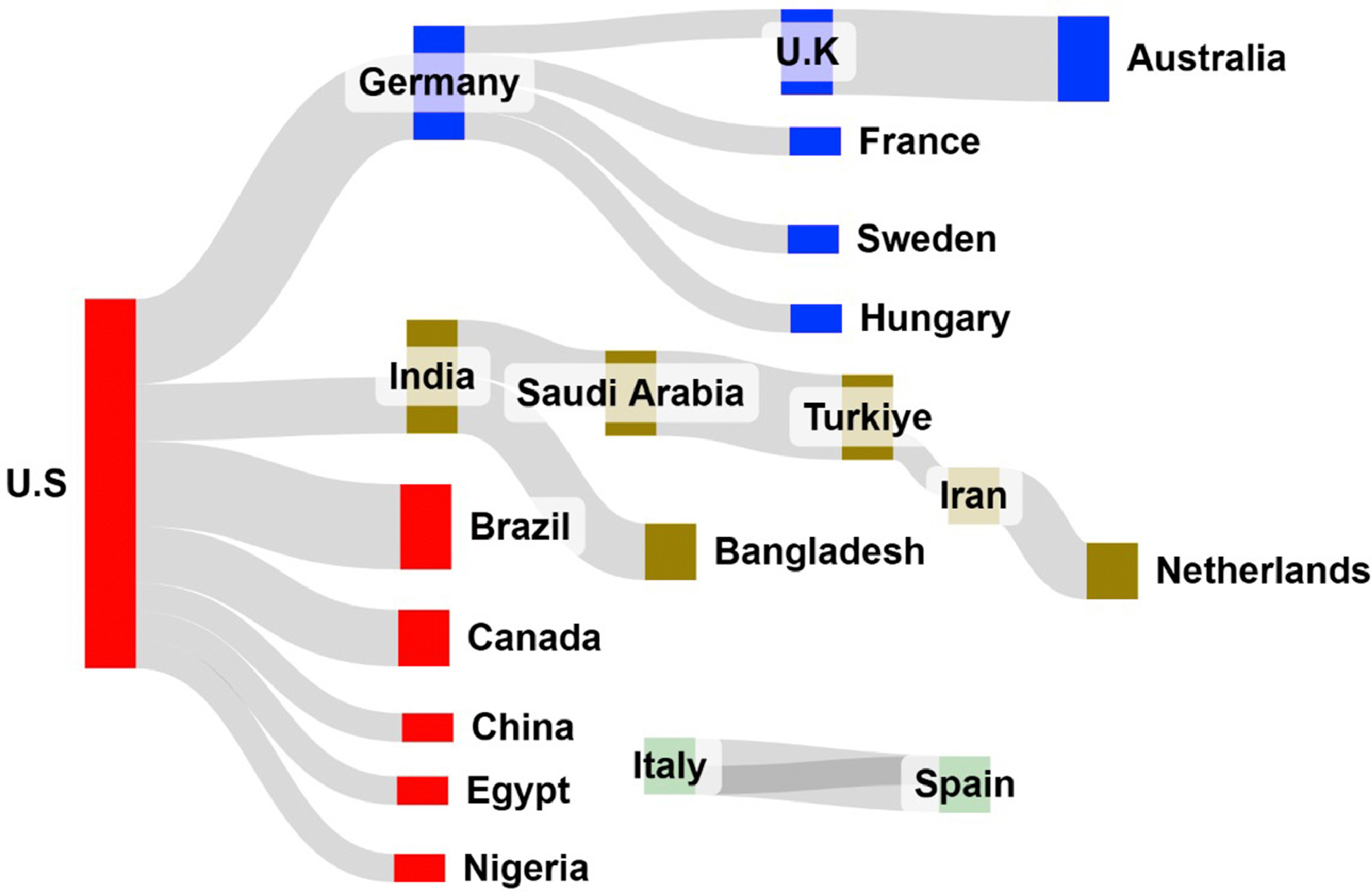

Supplement: Supplementary file 1 [file mmc1.jpg]
