# Supplementary material for: SankeyNetwork: A clear and concise visualization tool for bibliometric data
Source: MethodsX. 2025 Jun 3;14:103379. doi: 10.1016/j.mex.2025.103379 (PMC12179738; doi:10.1016/j.mex.2025.103379)
Supplement: Supplementary file 3 [file mmc3.pdf]

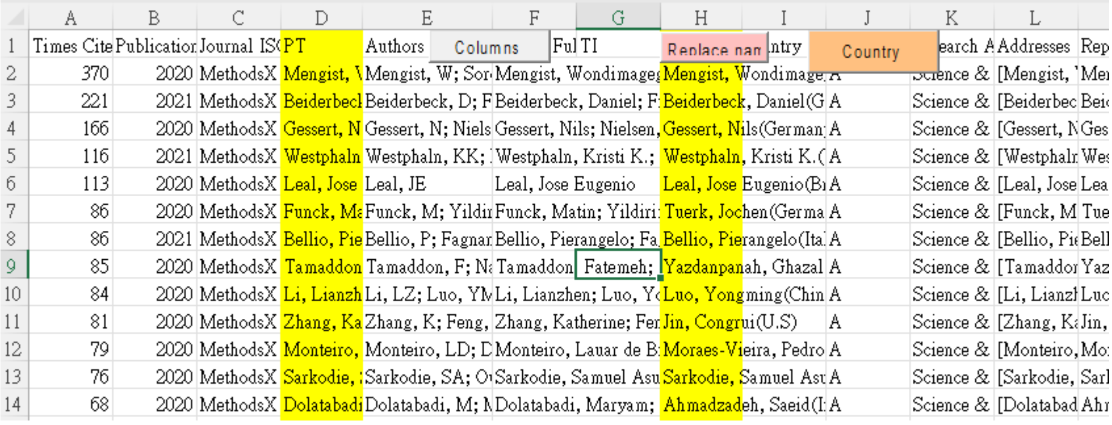

| | A | B | C | D | E | F | G | H | I | J | K | L | |
|---|---|---|---|---|---|---|---|---|---|---|---|---|---|
| 1 | Times Cite | Publication | Journal IS( | PT | Authors | Columns Ful | TI | Replace nam | ntry | Country | earch A | Addresses | Rep |
| 2 | 370 | 2020 | MethodsX | Mengist, \ | Mengist, W; Sor | Mengist, Wondimageg | | Mengist, Wondimage | | A | Science & | [Mengist, ' | Mer |
| 3 | 221 | 2021 | MethodsX | Beiderbeck | Beiderbeck, D; F | Beiderbeck, Daniel; F | | Beiderbeck, Daniel(G | | A | Science & | [Beiderbec | Bei |
| 4 | 166 | 2020 | MethodsX | Gessert, N | Gessert, N; Niels | Gessert, Nils; Nielsen, | | Gessert, Nils(German | | A | Science & | [Gessert, N | Ges |
| 5 | 116 | 2021 | MethodsX | Westphaln | Westphaln, KK; | Westphaln, Kristi K.; | | Westphaln, Kristi K.( | | A | Science & | [Westphaln | Wes |
| 6 | 113 | 2020 | MethodsX | Leal, Jose | Leal, JE | Leal, Jose Eugenio | | Leal, Jose Eugenio(B | | A | Science & | [Leal, Jose | Lea |
| 7 | 86 | 2020 | MethodsX | Funck, Ma | Funck, M; Yildir | Funck, Matin; Yildiri | | Tuerk, Jochen(Germa | | A | Science & | [Funck, M | Tue |
| 8 | 86 | 2021 | MethodsX | Bellio, Pie | Bellio, P; Fagnar | Bellio, Pierangelo; Fa | | Bellio, Pierangelo(Ita | | A | Science & | [Bellio, Pie | Bell |
| 9 | 85 | 2020 | MethodsX | Tamaddon | Tamaddon, F; Na | Tamaddon | Fatemeh; | Yazdanpanah, Ghazal | | A | Science & | [Tamaddon | Yaz |
| 10 | 84 | 2020 | MethodsX | Li, Lianzh | Li, LZ; Luo, YM | Li, Lianzhen; Luo, Yo | | Luo, Yongming(Chin | | A | Science & | [Li, Lianzh | Luo |
| 11 | 81 | 2020 | MethodsX | Zhang, Ka | Zhang, K; Feng, | Zhang, Katherine; Fen | | Jin, Congrui(U.S) | | A | Science & | [Zhang, Ka | Jin, |
| 12 | 79 | 2020 | MethodsX | Monteiro, | Monteiro, LD; D | Monteiro, Lauar de B | | Moraes-Vieira, Pedro | | A | Science & | [Monteiro, | Mo |
| 13 | 76 | 2020 | MethodsX | Sarkodie, | Sarkodie, SA; O | Sarkodie, Samuel Asu | | Sarkodie, Samuel Asu | | A | Science & | [Sarkodie, | Sar |
| 14 | 68 | 2020 | MethodsX | Dolatabadi | Dolatabadi, M; M | Dolatabadi, Maryam; | | Ahmadzadeh, Saeid(I | | A | Science & | [Dolatabad | Ahr |

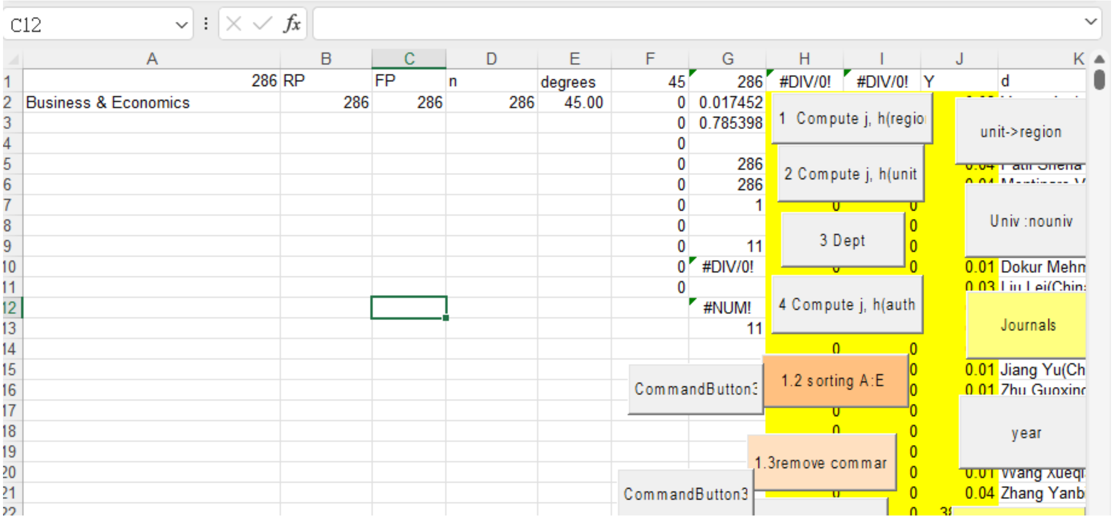

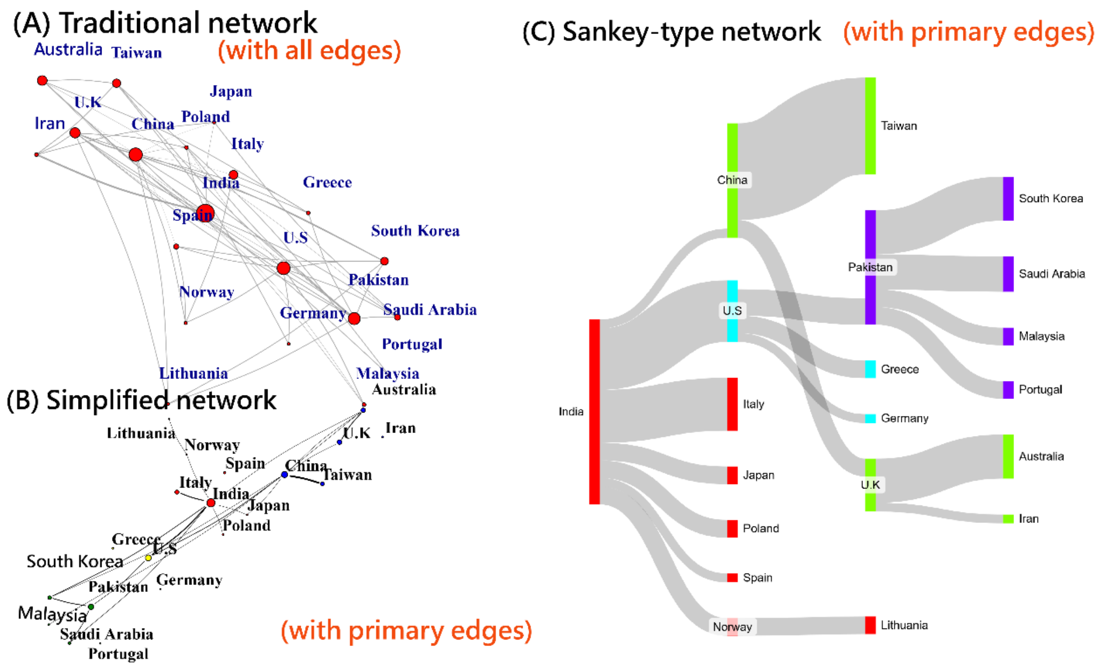


Figure 1 Networks in comparison with three types of visualizations

# Top 10 elements in each entity (n=2,252; h=33)

| Entity | RP | FP | n | h | Entity | | n | h |
|---|---|---|---|---|---|---|---|---|
| Country AAC=0.49 | | | | | Journal | AAC= | | IF |
| U.S | 296 | 292 | 302 | 14 | MethodsX | Q2\|61/134 | 2252 | 1.6 |
| India | 190 | 197 | 198 | 12 | | | | |
| Germany | 118 | 117 | 124 | 13 | | | | |
| China | 98 | 102 | 102 | 8 | | | | |
| Canada | 76 | 76 | 78 | 9 | | | | |
| Brazil | 74 | 76 | 77 | 9 | | | | |
| U.K | 67 | 75 | 75 | 8 | | | | |
| Italy | 71 | 71 | 74 | 10 | | | | |
| Spain | 70 | 70 | 73 | 9 | | | | |
| Indonesia | 72 | 72 | 72 | 6 | | | | |
| Institute AAC=0.55 | | | | | Year | AAC=0.47 | | h |
| Symbiosis Int Deemed Univ(India) | 16 | 14 | 18 | 3 | | 2024 | 570 | 8 |
| Shahid Bahonar Univ Kerman(Iran) | 14 | 13 | 15 | 5 | | 2023 | 525 | 13 |
| Inst Teknol Sepuluh Nopember(Indonesia) | 15 | 14 | 15 | 2 | | 2020 | 427 | 27 |
| South Asian Univ(India) | 11 | 12 | 13 | 3 | | 2021 | 408 | 20 |
| Univ Pannonia(Hungary) | 12 | 11 | 12 | 3 | | 2022 | 319 | 12 |
| McGill Univ(Canada) | 10 | 10 | 10 | 4 | | 2025 | 3 | |
| Univ Sains Malaysia(Malaysia) | 10 | 9 | 10 | 2 | | | | |
| Symbiosis Inst Technol(India) | 9 | | 9 | 1 | | | | |
| Texas A&M Univ(U.S) | 8 | 8 | 8 | 3 | | | | |
| Tech Univ Munich(Germany) | 8 | 8 | 8 | 4 | | | | |
| Departmen AAC=0.52 | | | | | Article type | AAC= | | h |
| Math | 44 | 36 | 47 | 6 | Article | | 2200 | 31 |
| Chem | 31 | 35 | 37 | 6 | Review | | 52 | 10 |
| Mech Engn | 24 | 28 | 30 | 6 | | | | |
| Civil Engn | 17 | 27 | 28 | 7 | | | | |
| Stat | 18 | 17 | 18 | 3 | | | | |
| Biol | 16 | 15 | 17 | 4 | | | | |
| Civil & Environm Engn | 14 | 16 | 17 | 3 | | | | |
| Chem Engn | 10 | 15 | 16 | 5 | | | | |
| Biol Sci | 8 | 15 | 16 | 5 | | | | |
| Phys | 14 | 15 | 15 | 4 | | | | |
| Author AAC=0.44 | | | | | Research area | AAC= | | h |
| Han, Fengxiang X.(U.S) | 5 | | 5 | 2 | Science & Technology - Other Topics | | 2252 | 33 |
| Budiantara, I. Nyoman(Indonesia) | 5 | | 5 | 2 | | | | |
| Humphries, Usa Wannasingha(Thailand) | 4 | | 4 | 2 | | | | |
| Sapnken, Flavian Emmanuel(Cameroon) | 4 | 4 | 4 | 2 | | | | |
| Yao, Qiang(U.S) | 4 | 2 | 4 | 2 | | | | |
| Hauer, Bernhard(Germany) | 4 | | 4 | 2 | | | | |
| Ibrahim, Rabha W.(Turkiye) | 4 | | 4 | 2 | | | | |
| Hassan, Jalal(Iran) | 4 | 3 | 4 | 1 | | | | |
| Otok, Bambang Widjanarko(Indonesia) | 4 | 1 | 4 | 1 | | | | |
| Yukhymenko-Lescroart, Mariya A.(U.S) | 4 | 4 | 4 | 1 | | | | |
| Keyword AAC=0.46 | | | | | Article | AAC=0.54 | | citation |
| MODEL | | | 29 | 12 | Mengist, Wondimagegn(Ethi | MethodsX | 2020 | 370 |
| WATER | | | 24 | 10 | Beiderbeck, Daniel(German | MethodsX | 2021 | 221 |
| IDENTIFICATION | | | 20 | 6 | Gessert, Nils(Germany) | MethodsX | 2020 | 166 |
| GROWTH | | | 18 | 6 | Westphaln, Kristi K.(U.S) | MethodsX | 2021 | 116 |
| SYSTEM | | | 16 | 6 | Leal, Jose Eugenio(Brazil) | MethodsX | 2020 | 113 |
| PERFORMANCE | | | 14 | 6 | Funck, Matin(Germany) | MethodsX | 2020 | 86 |
| DESIGN | | | 13 | 4 | Bellio, Pierangelo(Italy) | MethodsX | 2021 | 86 |
| QUALITY | | | 13 | 5 | Tamaddon, Fatemeh(Iran) | MethodsX | 2020 | 85 |
| BEHAVIOR | | | 11 | 5 | Li, Lianzhen(China) | MethodsX | 2020 | 84 |
| EXTRACTION | | | 9 | 7 | Zhang, Katherine(U.S) | MethodsX | 2020 | 81 |

$AAC=\gamma/(1+\gamma)$

$\gamma=(r1/r2)/(r2/r3)$ https://rasch.org/rmt/rmt263c.htm

h-index=33

Figure 2 Performance sheet for top 10 elements in each entity

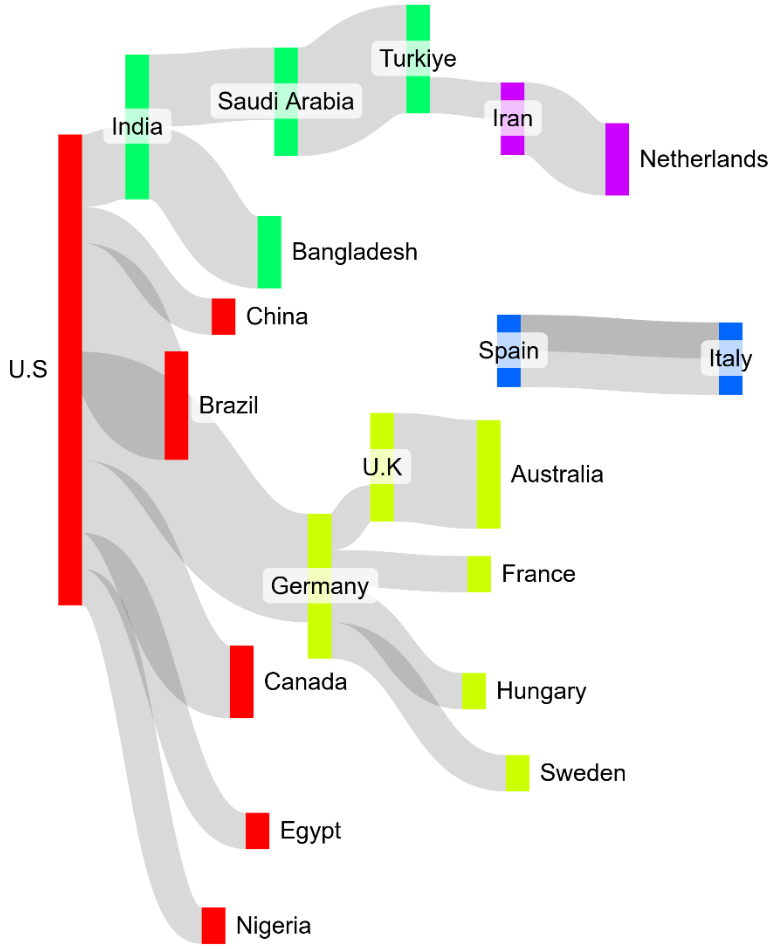


Figure 3 Coword analysis for top 20 elements, countries, institutes, and keywords

# Significant elements extracted from data

(adj.p.value<0.05 & |log2 FC|>1.0)

## (A)Volcano Plot

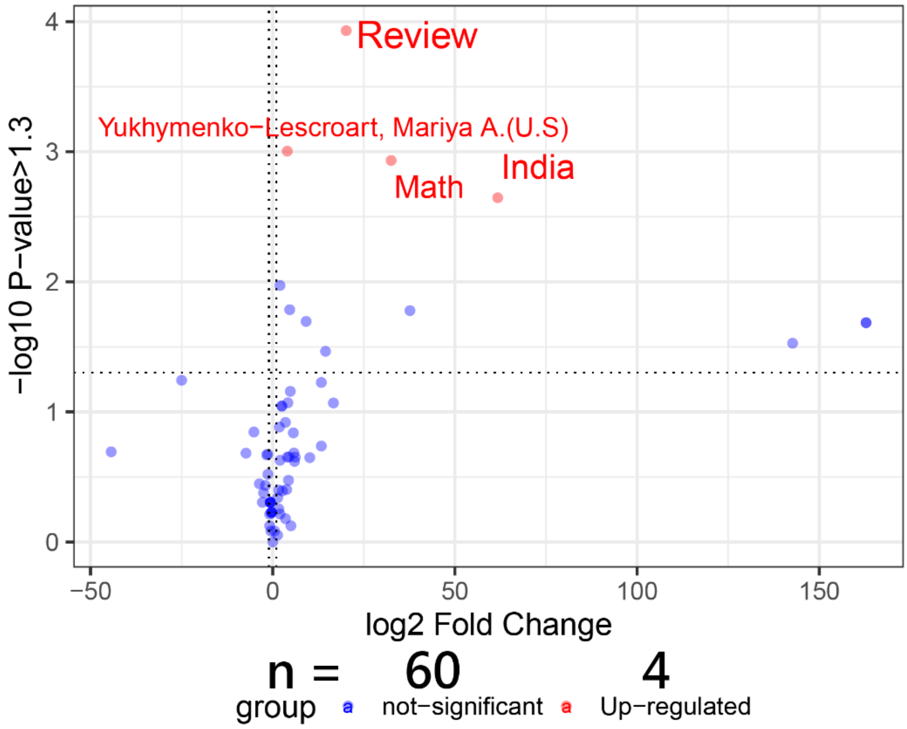


## (B)Heatmap

Phase I II

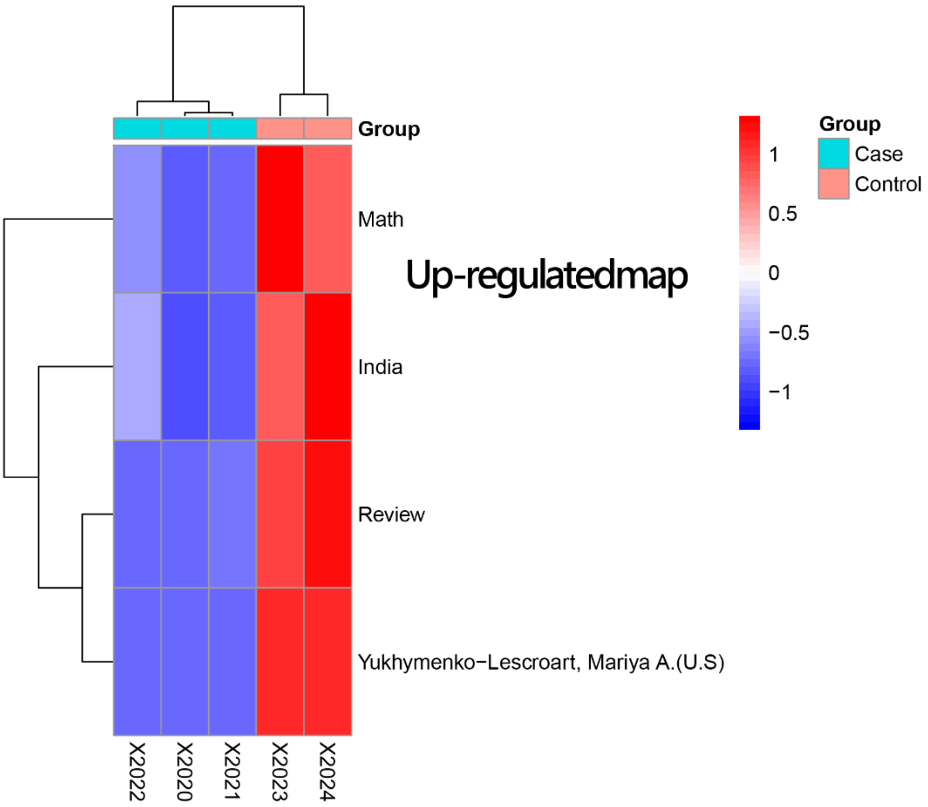

Figure 4 Significant elements with growth by count over years

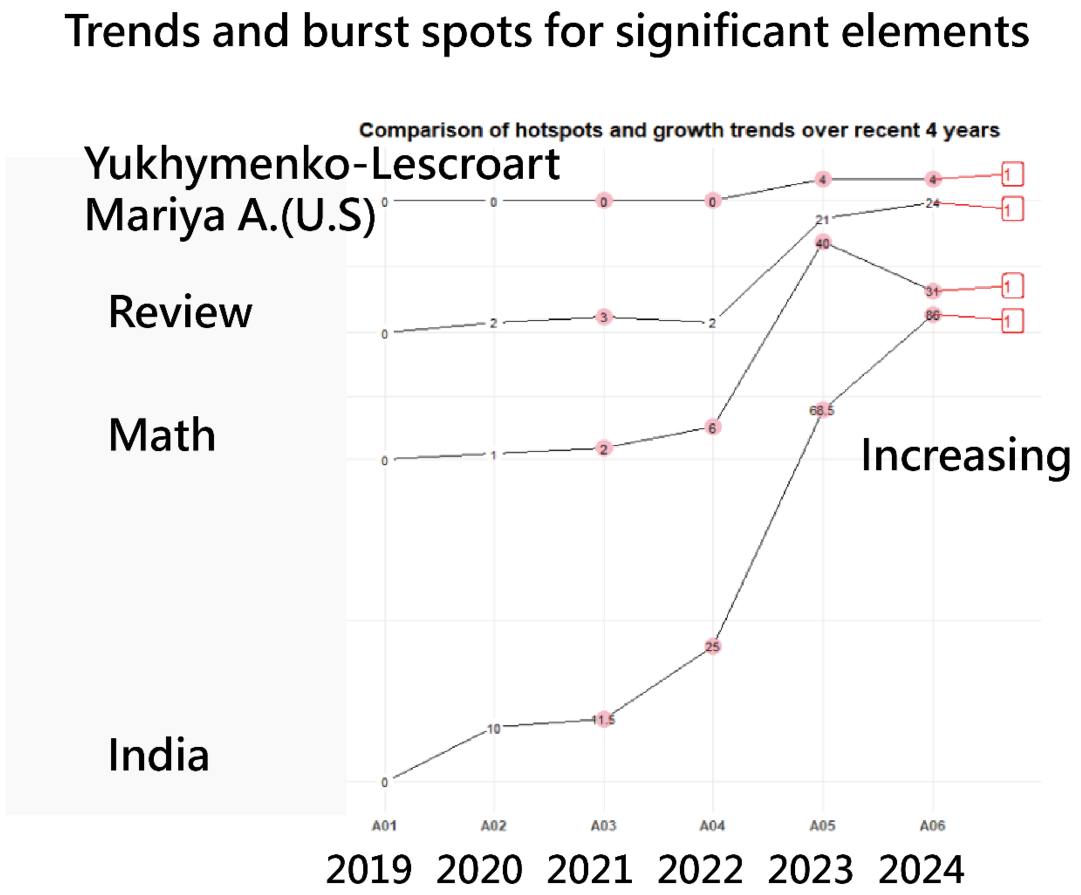


Figure 5 Analysis of growth trends for significant elements over years

https://www.raschonline.com/raschonline/cbpabc.asp

@@@ Volcano P.Value & adj.P.Val in R

@@@ 22B GEO Probe from GSE or data i

Submit

Tips for R

Copy & Paste the Code in R to Rstudio

Select text

```
col_names <- colnames(merged_data)

# Identify the positions of 'Gene', 'logFC', and 'P.Value'
gene_pos <- which(col_names == "Gene")
logFC_pos <- which(col_names == "logFC")
pvalue_pos <- which(col_names == "P.Value")

# Create a new order for the columns
# Place 'logFC' and 'P.Value' right after 'Gene'
new_order <- c(
    col_names[1:gene_pos],          # Columns up to 'Gene'
    col_names[c(logFC_pos, pvalue_pos)], # Move 'logFC' and 'P.Value' next
    col_names[(gene_pos + 1):(logFC_pos - 1)] # Remaining columns excluding the moved ones
)

# Reorder the dataframe columns
merged_data <- merged_data[, new_order]
colnames(merged_data)[colnames(merged_data) == "P.Value"] <- "log10P"
merged_data
```

```
rownames(expr_data)]
# Ensure that the annotation data frame is aligned with the
columns of expr_data
annotation_col <- data.frame(Group = colData$Condition)
rownames(annotation_col) <- colnames(expr_data[valid_top_gene_ids
, ])

# Draw the heatmap
pheatmap(expr_data[valid_top_gene_ids, ],
         annotation_col = annotation_col,
         cluster_rows = TRUE,
         cluster_cols = TRUE,
         show_colnames = TRUE,
         show_rownames = TRUE,
         scale = "row",
         color = colorRampPalette(c("blue", "white", "red"))(40))


expr_data_filtered <- expr_data[rownames(expr_data) %in%
df_sorted$ID, ]
```

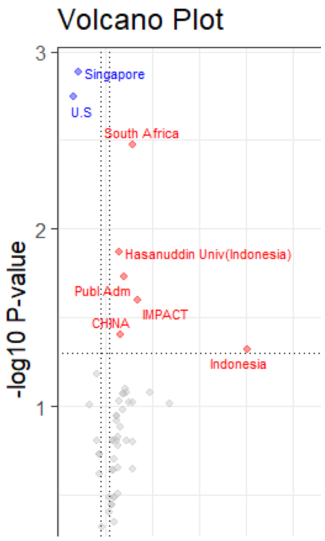

```
30
U.S -6.00
31                    Univ Pad
jadjaran(Indonesia)   3.50
          log10P  A2019  A2020  A
2021  A2022  A2023  A2024
1  2.866213e-04     0      0
0     0     5     4
2  5.243960e-03     0      0
0     0     3     2
3  2.591418e-02     0      1
0     0     2     7
4  5.436805e-05    17     15
14    17    85   115
5  5.099488e-05    18     16
18    18    92   124
6  1.011735e-02     0      0
0     0     5     2
7  3.609622e-06     2      4
2     2    18    17
8  7.711894e-04     0      0
```

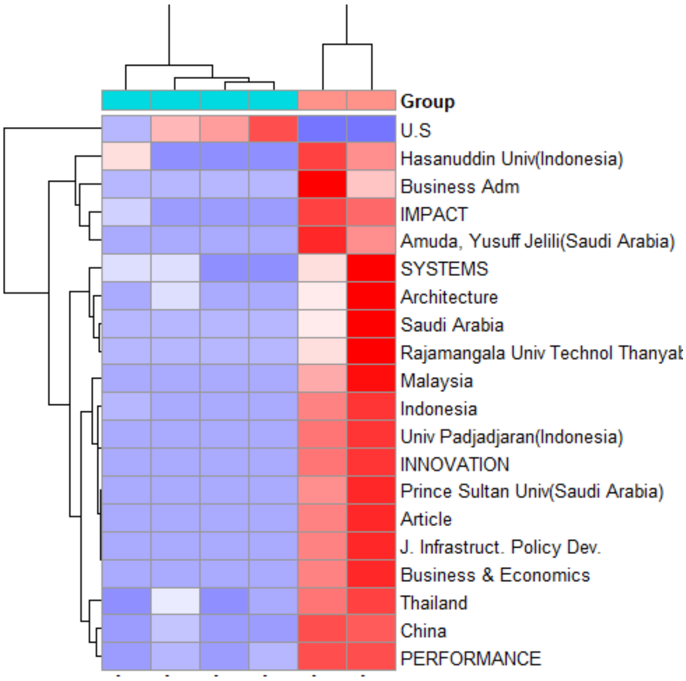


====to increase AUC-ROC if it deleted=

B***. AUC-ROC to reduce more Genes

@@@22G3 Loop AUC if genes deleted

Submit

Tips for R

Copy & Paste the Code in R to Rstudio

Select text

```
#

if (!requireNamespace("GEOquery", quietly = TRUE)) install.packages("GEOquery")
if (!requireNamespace("limma", quietly = TRUE)) install.packages("limma")
if (!requireNamespace("annotate", quietly = TRUE)) install.packages("annotate")

library(GEOquery)
```

| | A | B | C | D | E | F | G | H | |
|---|---|---|---|---|---|---|---|---|---|
| 1 | Gene | | | Times Cite | Publicatior | Journal ISC | PT | LA | D |
| 2 | Accounting | | | 84 | 2019 | | | | |
| 3 | Amuda, Yusuff Jelili(Saudi Arabia) | | | 17 | 2020 | | | | |
| 4 | Architecture | | | 13 | 2023 | | | | R |
| 5 | Business Adm | | | 13 | 2019 | | | | |
| 6 | China | | | 10 | 2022 | China | China | | |
| 7 | CHINA | | | 10 | 2021 | Review | U.S | U.S | R |
| 8 | | | | 9 | 2022 | | | | |
| 9 | onesia) | | | 8 | 2022 | Review | U.S | U.S | R |
| 10 | | | | 8 | 2020 | China | China | | |
| 11 | | | | 8 | 2020 | | | | |
| 12 | INNOVATION | | | 8 | 2019 | China | China | | |
| 13 | Malaysia | | | 7 | 2022 | | | | |
| 14 | M | | | 6 | 2023 | China | China | Management | M |
| 15 | M | | | 6 | 2020 | Thailand | Architecture | | |
| 16 | M | | | 5 | 2022 | South Afric | South Africa | | |
| 17 | P | | | 5 | 2021 | | | | |
| 18 | PERFORMANCE | | | 5 | 2021 | U.S | U.S | | |
| 19 | P | | | 5 | 2021 | Review | Review | | |
| 20 | P audi Arabia) | | | 5 | 2019 | | | | |
| 21 | P | | | 4 | 2023 | | | | |
| 22 | Rajamangala Univ Technol Thanyaburi(Thailand) | | | 4 | 2023 | Indonesia | Indonesia | | |
| 23 | Review | | | 4 | 2023 | | | | |

shieft top 10

A1 without remova

Remove space

Prepare network chart

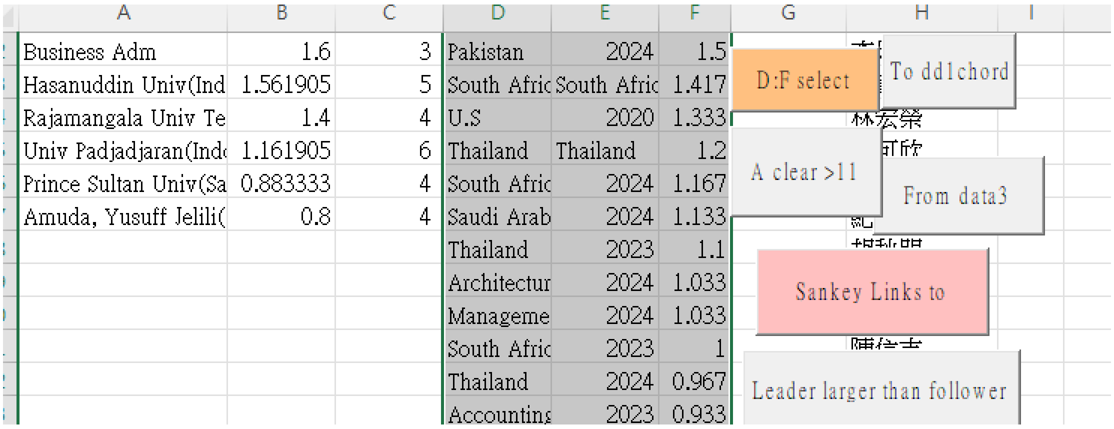


```
# Load necessary libraries
# Load necessary libraries
library(caret)
library(ggplot2)
library(e1071)    # For SVM
library(gridExtra)
library(dplyr)
library(tidyr)
library(pROC)
# Load the data
df <- read.table(textConnection('
Sample	Group	Accounting	Amuda, Yusuff Jelili(Saudi Arabia)
	Architecture	Business Adm	China	CHINA	Civil Engn
	Hasanuddin Univ(Indonesia)	IMPACT	Indonesia	INNOVATION
	Malaysia	Management	MANAGEMENT	MODEL	Pakistan
	PERFORMANCE	POLICY	Prince Sultan Univ(Saudi Arabia)	Publ Adm
	Rajamangala Univ Technol Thanyaburi(Thailand)	Review	Saudi Arabia
	South Africa	SYSTEMS	Thailand	U.S	Univ Padjadjaran(Indonesia)
Patient	Patient	2	1	1	2	9	2	2	2	5	18	1	7	3	4
	2	2	5	1	1	3	1	3	2	3	1	4	1	1
logFC	logFC	4.5	2.5	4.25	3.5	15	4.25	5.5	3	9	42.5	3.5	20.5	6.5
	7.75	5.5	5.5	9.5	5	2.5	4.5	4	6.75	9	6	3	8	-6	3.5
log10P	log10P	0.000286621	0.00524396	0.025914184	0.010117347
	3.61E-06	0.000771189	0.004415841	0.013543991	6.02E-05	3.42E-05
```

```
	0.001043968	0.001136054	3.99E-05	9.05E-05	9.86E-05	0.001236168
	8.25E-06	0.005481192	0.00524396	0.003897376	0.014336144
	0.001080681	0.015298631	0.006975389	0.025082794	0.000556412
	0.022273157	0.001043968
A2019	Case	0	0	0	0	2	0	0	0	0	0	0	0	0	0
	0	0	0	0	0	0	0	0	0	0	0	0	9	0
A2020	Case	0	0	1	0	4	0	1	0	0	0	0	0	0	0
	0	0	1	0	0	0	0	0	0	0	1	3	8	0
A2021	Case	0	0	0	0	2	0	0	0	0	0	0	0	0	1
	0	0	1	0	0	0	0	2	0	0	0	1	11	0
A2022	Case	0	0	0	0	2	1	1	2	2	4	0	0	0	0
	0	0	0	0	0	2	0	1	0	4	1	0	4	0
A2023	Control	5	3	2	5	18	4	4	4	10	37	3	14	6	9
	5	4	10	3	2	6	2	6	4	6	2	8	2	3
A2024	Control	4	2	7	2	17	5	8	3	9	50	4	27	7	7
	6	7	10	7	3	4	6	9	14	8	5	10	2	4
'), header = TRUE, sep = "\t", stringsAsFactors = TRUE)
#df <- read.csv("F:/RR/denmentiapredict.csv")
# Remove rows with logFC and log10P
patient_data_raw <- df[df$Sample == "Patient", ]

df3<-as.data.frame(df)
df3<-df[df$Sample == "logFC" | df$Sample == "log10P", ] # for "logFC", "log10P"
patient_data_raw
df3<-as.matrix(df3[,3:ncol(df)])
df3<-t(df3)
colnames(df3)<-c("logFC","log10P")
LogFC<-df3[,1]

df <- df[!(df$Sample %in% c("logFC", "log10P", "Patient")), ]
expr_data<-df[,3:ncol(df)]
expr_data<-t(expr_data)

rownames(expr_data)<-colnames(df[,3:ncol(df)])
colnames(expr_data)<-df$Sample
rownames(expr_data)
colnames(expr_data)
expr_data<-as.data.frame(expr_data)
```

```
expr_data$LogFC<-LogFC
# Create a data frame with sample labels
colData <- data.frame(SampleID =df$Sample,
                      Condition =df$Group)
# Convert the Condition column to a factor
colData$Condition <- factor(colData$Condition, levels = c("Case", "Control"))
colData$Condition
# Ensure Group is a factor
df$Group <- factor(df$Group, levels = c("Case", "Control"))

# Define the gene features for analysis (adjust based on confirmed column
names)
#genefeature <- c("ATP1B3", "BEX3", "EIF3C", "EIF3E", "LGALS3", "NOP53", "SGK1",
"XIST.1")
colnamedf <- colnames(df)
colnamedf <- colnamedf[-c(1, 2)]
#genefeature <- c("ATP1B3", "BEX3", "EIF3C", "EIF3E", "LGALS3", "NOP53", "SGK1",
"XIST.1")
genefeature <-colnamedf
# Normalize the data using training data scaling parameters
scaling_parameters <- preProcess(df[, genefeature], method = c("center",
"scale"))
df[, genefeature] <- predict(scaling_parameters, df[, genefeature])

# Split the data into training and test sets
set.seed(123)
trainingIndex <- createDataPartition(df$Group, p = 0.8, list = FALSE)
trainingData <- df[trainingIndex, ]
testData <- df[-trainingIndex, ]

# Train an SVM model (focus only on the gene features)
model <- train(Group ~ ., data = trainingData[, c(genefeature, "Group")], method
= "svmLinear", trControl = trainControl(method = "cv", number = 5))

# Normalize patient data using the same scaling parameters
# patient_data_raw <- data.frame(COL6A5 = 2.554, CPB1 = 2.629, ENPP6 =
1.877, MFSD4AAS1 = 2.302, PLA2G1B = 1.338, PROKR2 = 2.810)
```

```
patient_data_scaled <- predict(scaling_parameters, patient_data_raw)

# Predict the group for the normalized patient data
predicted_group <- predict(model, patient_data_scaled)
print(paste("as:", predicted_group))
caseorcontrol<-paste("Classified as:", predicted_group)


# Plot the PCA-transformed training data with the patient point highlighted
pca_model <- prcomp(trainingData[, genefeature], center = TRUE, scale. = TRUE)
trainingData_pca <- data.frame(pca_model$x[, 1:2], Group =
trainingData$Group)

# Add the patient point to the PCA plot
patient_pca <- predict(pca_model, newdata = patient_data_scaled)
trainingData_pca <- rbind(trainingData_pca, data.frame(PC1 = patient_pca[1, 1],
PC2 = patient_pca[1, 2], Group = "Patient"))

# Plot
svmplot <- ggplot(trainingData_pca, aes(PC1, PC2, color = Group, shape =
Group)) +
    geom_point(size = 3) +
    geom_point(data = trainingData_pca[trainingData_pca$Group ==
"Patient", ], color = "black", size = 4, shape = 15, fill = "black") +
    geom_text(data = trainingData_pca[trainingData_pca$Group == "Patient", ],
aes(label = caseorcontrol), vjust = -1, color = "black") +
    scale_color_manual(values = c("Case" = "red", "Control" = "green", "Patient"
= "black")) +   # Assign colors manually
    labs(title = "SVM Classification on PCA Components") +
    theme_minimal()
dev.off()   # Closes any open graphics devices
plot.new() # Start a new plot
print(svmplot)   # Re-plot your svmplot
# Print the plot
print(svmplot)
# Initialize columns in merged_df to store the AUC and confidence intervals
auc <- 0
CI1 <- 0
```

```
CI2 <- 0
count <- 0

# Start the loop for each gene
#for (i in 1:nrow(expr_data)) {

aldob_expr <-    expr_data

nrow(aldob_expr)
# Check if there are any corresponding rows for the gene
# if (nrow(aldob_expr) > 0) {

# Ensure colData is aligned with the SampleID columns
relevant_cols <- intersect(colnames(aldob_expr), colData$SampleID)
length(relevant_cols)
# if (length(relevant_cols) > 0) {
# Transform to long form data for SampleID columns
df_long <- aldob_expr %>%
    pivot_longer(
        cols = all_of(relevant_cols),
        names_to = "SampleID",
        values_to = "score"
    )

# Merge with the sample information in colData
adf <- merge(df_long, colData, by = "SampleID", all.x = TRUE)

# Reverse the group labels only when logFC < 0

adf <- adf %>%
    mutate(
        Condition = ifelse(LogFC < 0,
                           ifelse(Condition == "Case", "Control",
"Case"),Condition)
    )
adf$Condition <- ifelse(adf$Condition == "Control", 0, adf$Condition)
adf$Condition <- ifelse(adf$Condition == "Case", 1, adf$Condition)
```

```
adf$Condition[adf$Condition == 2] <- 0

# Convert Condition to binary (1 for Case, 0 for Control)


adf$Condition <- ifelse(adf$Condition == "Case", 0,
                        ifelse(adf$Condition == "Control", 1, adf$Condition))



# Filter out any rows with NA in the Condition
adf <- adf %>%
    filter(!is.na(Condition)) %>%
    rename(group = Condition) %>%
    select(group, score)
unique(adf$group)
adf$group <- as.numeric(adf$group)
group_0_present <- any(adf$group == 0)
group_1_present <- any(adf$group == 1)
group_1_present
group_0_present
# Step 4: Add a record with group = 0 and score = 0 if no 0 is present in df$group
if (!group_0_present) {
    adf <- rbind(adf, data.frame(score = 0, group = 0))
}

# Step 5: Add a record with group = 1 and score = mean(df$score if no 1 is
present in df$group
if (!group_1_present) {
    adf <- rbind(adf, data.frame(score = 1, group = 1))
}
# Check if df contains enough data points to compute AUC

if (length(unique(adf$group)) == 2) {    # Ensure there are both groups (0 and 1)
present

    # Calculate ROC curve and AUC
```

```
        roc_obj <- roc(adf$group, adf$score, levels = c(0, 1), direction = ">")
        auc_value <- round(auc(roc_obj), 3)

        if (auc_value<0.5){
            # Calculate ROC curve and AUC
            roc_obj <- roc(adf$group, adf$score, levels = c(0, 1), direction = "<")
            # Compute the AUC
            auc_value <- round(auc(roc_obj), 2)
        }

        # Compute the AUC
        auc_value <- round(auc(roc_obj), 3)

        # Compute the 95% confidence interval for the AUC
        ci_auc <- suppressWarnings(ci.auc(roc_obj))
        CI1 <- round(ci_auc[1], 3)
        CI2 <- round(ci_auc[3], 3)
        ci_auc
        auc_value
        # Store the results back in merged_df
        auc <- auc_value
        CI1 <- CI1
        CI2 <- CI2
        count <- nrow(aldob_expr)
    } else {
        # If there are not enough distinct groups, set AUC and CI values to 0
        auc <- 0
        CI1 <- 0
        CI2 <- 0
        count <- 0
    }
    # }
    #}
    #}
    count
    #Compute the AUC
    auc_value <- round(auc(roc_obj), 3)
    print(paste("AUC:", auc))
```

```
# Compute the 95% CI for the AUC
ci_auc <- ci.auc(roc_obj)
print(paste("95% CI for AUC:", round(ci_auc[1], 3), "-", round(ci_auc[3], 3)))

# Plot the ROC curve
plot(roc_obj, main = "ROC Curve", col = "blue", lwd = 2, ylim = c(0, 1))
legend("bottomright", legend = c(paste("AUC =", auc_value),
                                 paste("95% CI =", paste(round(ci_auc[1],
3), "-", round(ci_auc[3], 3)))),
       col = "blue", lwd = 2)
#write.csv(merged_df3, "F:/RR/merged_df3.csv", row.names = TRUE)


# Generate the ROC plot using ggplot2




roc_data <- data.frame(
    Specificity = rev(roc_obj$specificities),    # Reversing to match ggplot style
    Sensitivity = rev(roc_obj$sensitivities)
)

# Create the ROC plot using ggplot2
roc_plot <- ggplot(roc_data, aes(x = 1 - Specificity, y = Sensitivity)) +
    geom_line(color = "blue", size = 1) +
    labs(title = "ROC Curve", x = "1 - Specificity", y = "Sensitivity") +
    annotate("text", x = 0.2, y = 0.25, label = paste("Gene count =", count), color
= "blue", size = 5, hjust = 0) +

    annotate("text", x = 0.2, y = 0.2, label = paste("AUC =", auc_value), color =
"blue", size = 5, hjust = 0) +
    annotate("text", x = 0.2, y = 0.15, label = paste("95% CI =", round(ci_auc[1],
3), "-", round(ci_auc[3], 3)), color = "blue", size = 5, hjust = 0) +
    theme_minimal()

# Now combine the two ggplot objects side-by-side
grid.arrange(svmplot, roc_plot, ncol = 2)
```
